# CRISPR/Cas9 Genome Editing Technology: A Valuable Tool for Understanding Plant Cell Wall Biosynthesis and Function

**DOI:** 10.3389/fpls.2020.589517

**Published:** 2020-11-20

**Authors:** Yuan Zhang, Allan M. Showalter

**Affiliations:** ^1^Molecular and Cellular Biology Program, Ohio University, Athens, OH, United States; ^2^Department of Environmental & Plant Biology, Ohio University, Athens, OH, United States

**Keywords:** arabinogalactan-proteins, AGPs, CRISPR/Cas9, guide RNA, lignin, multiplexing, mutant, plant cell wall

## Abstract

For the past 5 years, clustered regularly interspaced short palindromic repeats/CRISPR-associated protein 9 (CRISPR/Cas9) technology has appeared in the molecular biology research spotlight. As a game-changing player in genome editing, CRISPR/Cas9 technology has revolutionized animal research, including medical research and human gene therapy as well as plant science research, particularly for crop improvement. One of the most common applications of CRISPR/Cas9 is to generate genetic knock-out mutants. Recently, several multiplex genome editing approaches utilizing CRISPR/Cas9 were developed and applied in various aspects of plant research. Here we summarize these approaches as they relate to plants, particularly with respect to understanding the biosynthesis and function of the plant cell wall. The plant cell wall is a polysaccharide-rich cell structure that is vital to plant cell formation, growth, and development. Humans are heavily dependent on the byproducts of the plant cell wall such as shelter, food, clothes, and fuel. Genes involved in the assembly of the plant cell wall are often highly redundant. To identify these redundant genes, higher-order knock-out mutants need to be generated, which is conventionally done by genetic crossing. Compared with genetic crossing, CRISPR/Cas9 multi-gene targeting can greatly shorten the process of higher-order mutant generation and screening, which is especially useful to characterize cell wall related genes in plant species that require longer growth time. Moreover, CRISPR/Cas9 makes it possible to knock out genes when null T-DNA mutants are not available or are genetically linked. Because of these advantages, CRISPR/Cas9 is becoming an ideal and indispensable tool to perform functional studies in plant cell wall research. In this review, we provide perspectives on how to design CRISPR/Cas9 to achieve efficient gene editing and multi-gene targeting in plants. We also discuss the recent development of the virus-based CRISPR/Cas9 system and the application of CRISPR/Cas9 to knock in genes. Lastly, we summarized current progress on using CRISPR/Cas9 for the characterization of plant cell wall-related genes.

## Introduction

**FIGURE 1:**
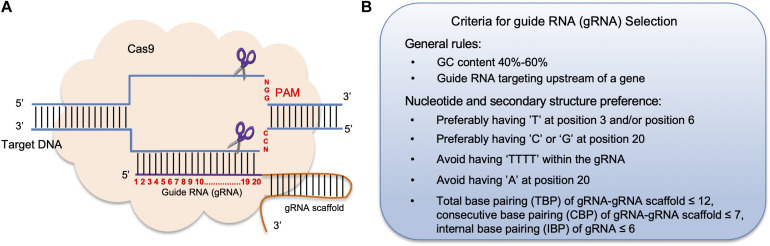
The principle of CRISPR/Cas9 mediated genome editing and criteria for guide RNA selection. **(A)** In the CRISPR/Cas9 system, a 20 nt guide RNA (gRNA) is complementary to the target DNA region in the host genome followed by a gRNA scaffold sequence. Each target DNA sequence ends with a protospacer adjacent motif (PAM), which is often the sequence “NGG.” The formation of the gRNA-DNA complex triggers the binding of the Cas9 endonuclease to the complex and generates a double-stranded break (DSB) 3 bp in front of the PAM. **(B)** General rules for choosing a gRNA sequence to improve its effectiveness.

In recent years, the clustered regularly interspaced short palindromic repeats (CRISPR) and CRISPR-associated protein 9 (Cas9) genome editing system has emerged as a versatile tool to perform precise gene targeting and mutations including gene insertions/deletions, gene replacements, and single base pair conversions (Zong et al., 2017; Soyars et al., 2018; Dong et al., 2020). CRISPR/Cas9 was first discovered as an adaptive immune defense system in bacterial cells as a mechanism to ward off foreign DNA (Jinek et al., 2012; Wiedenheft et al., 2012). When adapted for genome editing, the CRISPR/Cas9 machinery mainly contains two parts: a guide RNA (gRNA) and the Cas9 endonuclease. A gRNA is 20 nucleotides (nt) long and is a highly gene-specific sequence (Gao and Zhao, 2014). Each gRNA is complementary and binds to a specific target DNA sequence that ends with a short DNA sequence, known as the protospacer adjacent motif (PAM), which is often “NGG.” The PAM region is essential for Cas9 binding and is found 3 bp downstream of the cleavage site of the Cas9 endonuclease (Ran et al., 2013). Adjacent to the 3′ end of the 20 nt gRNA is an ∼80 nt long gRNA scaffold sequence that is necessary for Cas9 binding (Jiang and Doudna, 2017). Once the gRNA-Cas9 complex forms, Cas9 makes a double-strand cut exactly 3 bp before the PAM sequence (Jiang et al., 2013). The break site is mainly repaired by non-homologous end joining (NHEJ), which is often error-prone and results in insertion or deletion (indel) mutations at the cut site (Figure 1A). Such indel mutations often lead to frame-shift mutations, affecting protein translation and thereby disrupting a gene’s function. Plant scientists have begun to utilize CRISPR/Cas9 gene editing technology in both model plants and crop plants to manipulate genetic pathways, improve various agronomic traits, and produce pathogen-resistant crops (Gurumurthy et al., 2016; Li et al., 2018b; Makarova et al., 2018; Wang C. et al., 2019).

The plant cell wall is a dynamic and complex extracellular organelle that is essential for cell survival and has great economic value (Makarova et al., 2018). The primary cell wall consists of cellulose microfibrils embedded in an aqueous cell wall matrix, which is largely composed of two polysaccharides, pectin and hemicellulose, as well as some proteins (Cosgrove, 1997). The secondary cell wall is produced between the primary cell wall and the plasma membrane after cell expansion is completed. Approximately 40–50% of the secondary cell wall is made up of cellulose, followed by hemicellulose (xyloglucan and xylan), and lignin (Zhong and Ye, 2015). Approximately 10% of the cell wall is composed of proteins, mostly hydroxyproline-rich glycoproteins (HRGPs), which include arabinogalactan-proteins (AGPs), extensins (EXTs), and proline-rich proteins (PRPs) (Showalter, 1993).

In this review, we focus on the rationale and principles associated with using CRISPR/Cas9 constructs to generate genetic mutants that disrupt gene/proteins, which are associated with plant cell wall biosynthesis. We first discuss strategies to optimize CRISPR/Cas9 design, including choosing the best gRNA and Cas9 promoter. We then explore the multiplexing capacity of the CRISPR/Cas9 mediated gene editing system and its applications. Next, we describe CRISPR/Cas9 mutant generation and detection, as well as methods for Cas9-free mutant identification. And finally, we summarize current efforts utilizing CRISPR/Cas9 technology to elucidate gene functions related to plant cell wall biosynthesis.

## Optimization of gRNA Design in the CRISPR/Cas9 System

Several publicly available web-based tools such as CRISPR-PLANT^1^, E-CRISP^2^, CHOPCHOP^3^, Tefor^4^, and CRISPR-P 2.0^5^, are widely used for gRNA design. These design tools often provide a list of possible gRNA sequences and rank them by their targeting scores for any gene of interest in a given plant species (Moreno-Mateos et al., 2015). Depending on the gRNA sequence chosen, potential gRNA picks contain zero to many potential off-target sites with different off-target scores. It is noteworthy that the specificity of a gRNA sequence is mainly determined by the 8–12 nt gRNA sequence (i.e., the seed region) next to the PAM sequence, also known as the PAM-proximal region. As the structure of the RNA–DNA heteroduplex in the PAM-distal region is more flexible than the PAM-proximal region, the proximal region is nearly intolerable to any mismatches compared to the distal region (Jinek et al., 2012; Jiang and Doudna, 2017). Knowing the specificity of a gRNA sequence also helps to evaluate potential off-target effects, since off-target effects are less likely to occur when mismatches appear in the seed region.

The gRNA design websites also display other features, including locations of each gRNA, its GC content, restriction enzyme (RE) sites within the gRNA sequence, potential off-target genes and the corresponding off-target scores. Generally, one should select gRNA(s) that target the 5′ region of a gene to ensure that the translation of a functional protein is terminated as early as possible. Also, a functional gRNA(s) often contains 40–60% GC content in order to increase its binding affinity with the Cas9 protein (Samarut et al., 2016; Figure 1B). Besides using gRNA design tools, several other criteria for gRNA(s) selection should also be taken into consideration. One study found that high mutagenesis frequency is associated with having a “T” at position 3 and/or position 6, as well as a “C” at position 20 of a gRNA sequence, whereas having an “A” at position 20 lowered the gRNA targeting rate (Liu et al., 2016b). Moreover, gRNAs ending with “GG” can improve Cas9 enzyme activity up to 10-fold compared with gRNAs that ended with “AG” or “GA” (Gagnon et al., 2014). Another study found that having a “G” adjacent to the PAM sequence resulted in higher mutagenesis rates *in vitro* (Gagnon et al., 2014; Figure 1B). Furthermore, a gRNA that contains four or more consecutive “T” nucleotides should be avoided, as such sequences can be recognized as a transcription stop site (Ma and Liu, 2016; Figure 1B).

In fact, nucleotide compositions of a gRNA at different positions can influence the binding affinity and the structure of the Cas9-gRNA-DNA complex (Ma et al., 2015). Further analysis found that in order for a Cas9-gRNA-DNA to form a stable secondary structure, base-pairing rules between an individual a gRNA-gRNA scaffold have been established: gRNA-gRNA scaffold should have less than 12 total base-pairings, a gRNA-gRNA scaffold should have less than 7 consecutive base-pairings, and internal gRNA base-pairings should be less than 6 (Liang et al., 2016). Therefore, choosing gRNA(s) that meet these secondary structure criteria can greatly improve gene editing efficiency (Figure 1B).

## Optimization of Promoters Used in the CRISPR/Cas9 System

Apart from the nucleotide composition and secondary structure of a gRNA, the mutagenesis efficiency of CRISPR/Cas9 is also dependent on the vector system and whether the plant is a monocot or eudicot. In a CRISPR/Cas9 expression vector, the promoter(s) used to drive Cas9 expression plays a key role in the likelihood and types of mutations (i.e., chimeric or non-chimeric). Constitutive promoters such as the 35S promoter, 2 × 35S promoter, rice ubiquitin promoter, and ubiquitin promoters from different plant species are often used for gene editing in plants (Jiang et al., 2014; Zhang et al., 2014). For Arabidopsis gene editing, the 35S promoter, however, is not recommended because 35S-driven expression has low activity during embryogenesis and in germ-line cells when using floral dip transformation. Thus, the 35S promoter generates more somatic mutations and fewer mutations in the reproductive tissue, making the mutations less inheritable (Feng et al., 2018). The Arabidopsis ubiquitin (AtUBQ10) promoter, which is highly expressed during embryogenesis, is a better choice. Although the AtUBQ10 promoter improves the mutagenesis rate, the majority of CRISPR lines generated with the AtUBQ10 promoter are chimeric mutants, which often require one more generation (i.e., a T2 generation) to determine the exact mutation type (Feng et al., 2014; Yan et al., 2016).

Therefore, the expression of Cas9 should be high in the early developmental stages in order to obtain homozygous and stable mutants. Arabidopsis promoters including the egg-cell (E.C) specific promoters such as E.C 1.1 and E.C 1.2 (DD45), pollen-specific promoters such as LAT52, sporogenous cell specific promoters such as SPL, and the Yao promoter which is highly expressed both in the meristem and during embryogenesis can increase the chance of homozygous and heritable mutations in Arabidopsis (Wang Z.-P. et al., 2015; Yan et al., 2015; Mao et al., 2016).

## CRISPR/Cas9 Multiplexing Methods and Applications

**FIGURE 2:**
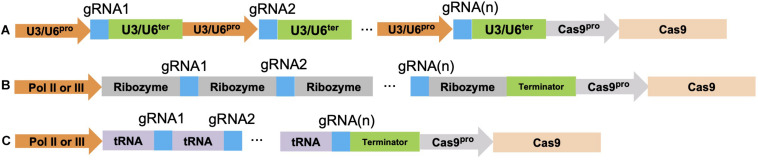
Three CRISPR/Cas9 multiplexing approaches. **(A)** Multiple gRNA(s) can be assembled together as multiple transcription cassettes. Either a U3 or U6 promoter/terminator is used depending on the monocot or dicot species being targeted. **(B)** CRISPR/Cas9 multiplexing is done by spacing a ribozyme sequence before and after each gRNA sequence; these ribozyme-gRNA-ribozyme (RGR) units undergo self-cleavage after transcription. **(C)** The polycistronic tRNA-gRNA (PTG) system fuses each gRNA with a tRNA sequence, endogenous RNaseP and RNaseZ can recognize, cleave at the tRNA sequence, and release the gRNA after transcription. The blue color indicates different gRNA sequences. Each gRNA multiplexing gene construct also contains a *Cas9* gene under the control of a specific promoter such as the actin, ubiquitin, 35S, and the germline cell promoter.

One useful extension of CRISPR-Cas9 is its multiplexing capacity. Currently there are no reliable ways to accurately predict the efficiency of a single gRNA *in vivo*. Consequently, to ensure successful gene editing, multiple gRNAs can be used to target different loci of a single gene simultaneously. The typical approach for CRISPR/Cas9 multiplexing is to assemble multiple gRNA transcription units in a head-to-tail fashion in a binary vector that contains a *Cas9* gene expression cassette. Each gRNA transcription unit contains an RNA polymerase (Pol) III promoter, such as the rice U3 or Arabidopsis U6 small nuclear RNA (snRNA) promoters, the gRNA, and the gRNA scaffold sequences followed by a U3 or U6 terminator sequence (Li et al., 2007; Figure 2A). The snRNAs are a class of genes that function in pre-mRNA splicing in both plants and animals. These U3 or U6 snRNA promoters are constitutively expressed and therefore are able to generate relatively high levels of an RNA transcript (Li et al., 2007). Based on this strategy, a number of convenient cloning vectors were developed that only require the insertion of the gRNA sequence(s) into the cassette for both monocot and dicot species. One published CRISPR-Cas9 construct incorporated 11 multiple cloning sites in the vector and can allow for the incorporation of 10 distinct gRNAs (Liang et al., 2016). Another research group engineered a vector that can include up to 12 different gRNAs for both monocot and dicot plants (Ma and Liu, 2016; Čermák et al., 2017). Golden Gate and Gibson cloning are two popular approaches used to facilitate CRISPR-Cas9 multiplexing cloning. Both methods use a Type II restriction endonuclease such as *Bsa*I and T4 or T7 ligase, which enables digestion and ligation of multiple gRNA cassettes in one chemical reaction. However, the U3 or U6 promoter requires an “A” or “G” at the transcription start site, which means the chosen gRNA should either start with such a nucleotide or have an extra “A” or “G” added to the 5′ end of the selected gRNA sequence.

Two other multiplexing approaches that use RNA Pol II or RNA Pol III for gRNA(s) transcriptions are the ribozyme-gRNA-ribozyme (RGR) system and the polycistronic tRNA-gRNA (PTG) system (Gao and Zhao, 2014; Xie et al., 2015; Figures 2B,C). Unlike RNA Pol III which is a constitutive promoter, RNA Pol II allows for cell or tissue specific expression (He et al., 2017). In the RGR system, a 5′ hammerhead ribozyme sequence cleavage site was designed immediately in front of the gRNA sequence, whereas another cleavage site from the hepatitis delta virus (HDV) ribozyme sequence was designed to occur at the 3′ end of the gRNA sequence (Figure 2B). Both ribozyme sequences undergo self-cleavage to release the individual gRNA once it is transcribed (He et al., 2017). In the PTG system, each gRNA is spaced by a tRNA sequence and up to eight different gRNAs (PTG units) can be assembled in this system (Figure 2C). Once a PTG construct is transcribed in the cell, RNase P and RNase Z recognize and cleave at the tRNA sequence, thus releasing the gRNA. This method was developed for targeting multiple genes, such as the mitogen-activated protein kinase (MPK) genes (*MPK1*, *MPK2*, *MPK5*, and *MPK6*) in rice (Xie et al., 2015; Minkenberg et al., 2017). By using the PTG targeting approach, single gRNA mutation efficiency varied from 13–100%. As for multi-gene targeting, 50% of the T0 transgenic lines contained mutations for all eight gRNAs targeting the 4 *MPK* genes (Xie et al., 2015).

Because the tRNA sequence in the PTG system is conserved across plant species, the PTG cloning vector is universal. Depending on the species to be targeted, these PTG units can be assembled into either a monocot or dicot CRISPR-Cas9 expression vector using the Golden Gate or Gibson cloning approach. The PTG system also allows for the expression of up to eight gRNAs in a single transcript given the small size of an individual gRNA-tRNA unit. To date this system has been used for multi-gene targeting in Arabidopsis, crop plants (rice, wheat, and *Brassica napus*), *Drosophila*, and human cell lines (Nissim et al., 2014; Port and Bullock, 2016; Qi et al., 2016; Yang H. et al., 2017). Exploitation of the CRISPR-Cas9 multiplexing capacity has made it possible to edit multiple genes simultaneously within one or two generations, as well as knock-out genes that are closely linked. It also opens up many possibilities for plant breeding. For example, this multiplexing capacity facilitates gene editing of polyploid crop species such as wheat, strawberry, *B. napus*, and *Camelina sativa* (Wang W. et al., 2016; Jiang et al., 2017; Yang L. et al., 2017). Moreover, it has been adopted to edit multiple quantitative trait loci (QTL) that control traits such as yield and kernel size in rice and maize (Shen et al., 2018; Zhou et al., 2019). More recently, a rapid *de novo* domestication has been successfully achieved in tomato using the CRISPR-Cas9 multiplexing approach that edited six “domestication genes” that controlled plant architecture, yield, and nutritional value in wild-type (WT) genomes, which resulted in tomatoes that possess both genetic diversity of the WT tomato along with modern tomato traits (Li et al., 2018a).

## CRISPR/Cas9 Mutant Generation, Detection, and Phenotypic Analyses

CRISPR-Cas9 knock-out mutants are different from T-DNA insertion mutants in the mutant generation process. For T-DNA insertion mutants, heterozygous mutants are first produced in the T0 or T1 generation and segregate into heterozygous and homozygous mutants after self-pollination in later generations. In the CRISPR-Cas9 system, when a DSB (double-stranded break) created by Cas9 occurs, the DSB could occur in one allele or both alleles of a target gene resulting in monoallelic (heterozygous) or biallelic (homozygous) mutants (Soyars et al., 2018). Additionally, chimeric/mosaic mutants are often generated when Cas9 is expressed in some (but not all) somatic cells. In other words, there is a higher chance to obtain non-chimeric mutations when Cas9 is expressed during embryogenesis or in germ line cells. Consequentially, rice and tomato, which use a callus-based transformation method, generally have a lower chance to produce chimeric/mosaic mutants in the T1 generation compared to Arabidopsis, which relies on floral dip transformation, and has a somewhat higher chance to produce chimeric/mosaic mutants in the T1 generation, especially when using constitutive promoters (Fauser et al., 2014). Furthermore, Cas9 can still generate DSBs in subsequent plant generations if it is not segregated out from the mutants (Feng et al., 2014).

**FIGURE 3:**
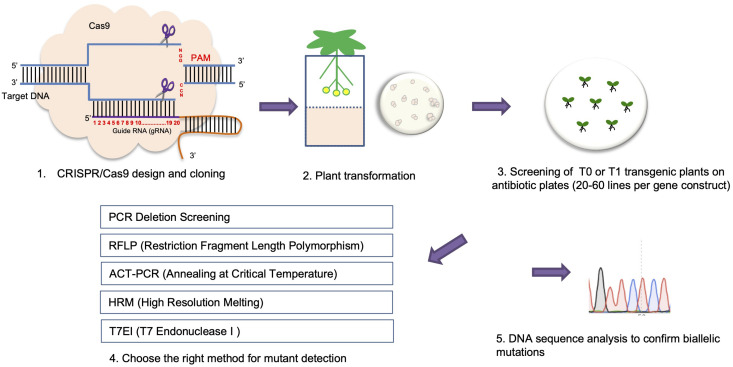
A pipeline for using CRISPR/Cas9 mediated genome editing to generate plant knock-out mutants.

Typically, CRISPR-Cas9 mediated mutations are 1 bp insertion/deletions (indels) in plants (Zhang et al., 2014; Ma et al., 2015; Svitashev et al., 2015; Xiong et al., 2019). Compared to T-DNA insertion mutant screening where the exact mutation type (i.e., homozygous or heterozygous mutants) can be easily detected by PCR, methods for detecting mutations for CRISPR mutant screening are generally more difficult and varied with one exception (Figure 3). This exception involves using two gRNAs targeting different loci of a gene, where a larger fragment deletion can occur in a CRISPR mutant that can be easily detected by PCR (i.e., PCR deletion screening) (Figure 3). However, such a large deletion event happens less frequently, compared to only one or the other gRNA working and generating an indel mutation.

Two widely used methods for CRISPR/Cas9 mutant screening are the T7 endonuclease I (T7EI) mismatch cleavage assay and RFLP (restriction fragment length polymorphism) analysis (Botstein et al., 1980; Vouillot et al., 2015). For both assays, a target gene region is first amplified by PCR. Both WT and homozygous mutants produce perfect matches after denaturing and re-annealing of the PCR product. Heterozygous mutants produce heteroduplexes, revealing indels that can be cleaved by T7EI and identified by DNA gel electrophoresis. To identify homozygous mutations, a second round of the T7E1 assay needs to be done. Here, DNA from homozygous mutants is mixed with WT DNA before the denaturing and re-annealing step. In this case, only homozygous mutants are able to form heteroduplexes with WT DNA and are cleaved by T7E1 (Figure 3). RFLP analysis is an ideal method to perform mutant screening when a RE recognition site resides within the gRNA sequence (Kim et al., 2014). In this assay, the appropriate RE is added after PCR amplification. Monoallelic or biallelic mutations will disrupt the RE cleavage site in either one or both mutant alleles, resulting in either one larger size band, or one larger and one smaller size band in the mutant compared with one smaller (duplet) band when using WT DNA for digestion after DNA gel electrophoresis (Figure 3).

One simple and accurate approach for detection of small indels is the high-resolution melting (HRM) curve assay, which relies on melting curves for indel mutation detection through a qPCR reaction (Wittwer et al., 2003). The HRM method begins by amplifying a 80–95 bp gene fragment including the target site in the presence of a florescent dye. At the last step of the PCR reaction, the PCR product undergoes a denaturation-annealling step (95°C, 30 s and 25°C, 30 s). This step allows for the formation of homoduplexes and heteroduplexes depending on mutation types. Next, a melting curve is generated during the denaturation step when the amplicon is denatured 65–95°C by increasing the temperature in 0.2°C increments. Since the fluorescent dye used by HRM only binds to double-stranded DNA, the denaturation step releases the fluorescent dye from the bound DNA. Any changes in the nucleotide sequence are shown by different melting temperature during the melting step resulting in different melting curves in this qPCR program (Simko, 2016; Figure 3).

Another recently developed method for CRISPR/Cas9 mutation screening is the ‘annealing at critical temperature PCR (ACT-PCR) method (Hua et al., 2017; Guo et al., 2018). This method is based on amplifying a gene fragment flanking the target site using two primers: a left primer ending with a 1–4 bp overlap with the DSB site, and a right primer located downstream of the target site. To employ this method, gradient PCR is first used to amplify WT DNA to determine the critical T_m_ for the gene target followed by amplifying mutants using the same T_m_. Any mismatches that occur in the mutants may prevent the primers from binding at the critical T_m_. This method, however, cannot differentiate chimeric, heterozygous, and homozygous mutations. To date, this ACT-PCR method has been applied in Arabidopsis, rice, cabbage, and zebrafish (Hua et al., 2017; Guo et al., 2018; Xiong et al., 2019; Figure 3).

Other methods also exist for CRISPR/Cas9 mutant analyses such as indel detection by amplicon analyses (IDAA), single-strand conformational polymorphism (SSCP) analyses, and an *in vitro* Cas9 cleavage assay developed by Clontech Laboratories (Zheng et al., 2016; Lonowski et al., 2017; Zhou et al., 2017). Although these other methods can also accurately detect indel mutations, these methods may require special equipment or reagents to perform the assay.

Potential mutants that are obtained by the above screening methods are then subjected to DNA sequence analysis, typically by Sanger sequencing. A target site that shows clear, single nucleotide peaks 3 bp in front of the PAM sequence on a sequencing chromatogram is an indication of a homozygous or biallelic mutation. However, CRISPR-Cas9 is likely to induce chimeric or heterozygous mutations, which are shown as double or overlapping nucleotide peaks in the sequencing chromatogram starting right from the gRNA binding site. In these cases, it is difficult to discern the exact mutation type from the chromatogram. Therefore, tools, such as DSdecode, were developed to generate sequencing results for both alleles from a mixed peak Sanger sequencing chromatogram (Liu et al., 2015). For even more accurate analysis, CRISPR-Cas9 induced mutations occurring in these cases can directly be quantified with respect to the percentages of each mutation using next generation sequencing (NGS) (Bell et al., 2014).

CRISPR mutant lines may show stronger phenotypes than actual homozygous mutant phenotypes in the T1 generation for unknown reasons (Soyars et al., 2018). Because of that, phenotypic traits shown in the early generation provide a rapid way to assess mutant phenotypes as well as narrow down mutants for screening. To obtain stable CRISPR/Cas9 mutant lines, it is important to differentiate true homozygous mutants from chimeric mutants. A Sanger sequencing result of a T1 mutant line sometimes can be misleading when it comes to a chimeric mutant as it may only indicate the genotype of the particular tissue being sequenced. To avoid tissue biases, one study extracted a pool of DNA from different organs for gene amplification (Schumacher et al., 2017). Another protocol suggested the collection of seeds from separate branches of a chimeric mutant to prevent segregation of a desired mutation in the next generation (Yan et al., 2016). In addition, progenies of the T1 mutant line should be confirmed to ensure stable transmission of a mutation.

## Cas9-Free (“T-DNA” Free) Mutant Screening

Because the presence of Cas9 in a mutant has the potential to generate subsequent cuts in the genome and create other gene mutations, it is necessary to screen for Cas9-free offspring once a mutant line is identified. The traditional way of using PCR to screen for Cas9-free mutants is labor-intensive and tedious. Two ingenious ways were developed for Cas9-free mutant screening that involves making fusion proteins with a functional Cas9 and exclusively expressing it in Arabidopsis seeds. One study fused mCherry with Cas9 under the control of the At2S3 seed-specific promoter (Gao et al., 2016). Another study fused Cas9 together with TagRFP under control of the Oleosin-1 (OLE1) seed oil body specific promoter (Shimada et al., 2010; Tsutsui and Higashiyama, 2017). In both methods, Cas9-free transgenic lines can be screened directly from T1 seeds using a fluorescent microscope, which has greatly lessened the workload and the timeframe for obtaining Cas9-free mutant lines. Similarly, by fusing the CRISPR construct with a GFP tag and by delivering this construct by hairy root transformation into *Brassica carinata*, successful transgenic lines were identified using a blue-green LED flashlight (Kirchner et al., 2017).

## Virus-Mediated CRISPR/Cas9 Gene Editing

Several plant viruses have been engineered and used as vectors to deliver CRISPR/Cas9 to generate knock-out mutants (Ali et al., 2015; Hu J. et al., 2019; Ellison et al., 2020; Ma et al., 2020). The virus-based method delivers a preassembled CRISPR/Cas9 construct into a specific tissue through injection. This approach provides an alternative to performing gene editing without tissue culture steps and allows for quick assessment of mutant phenotypes as well as multiplexing (Liu and Zhang, 2020). Moreover, it is possible to obtain DNA-free gene-edited plants as the DNA or RNA viruses are transiently expressed or do not have a DNA phase during replication; therefore, there is no incorporation of T-DNA into the host genome. Nevertheless, the application of a viral-based CRISPR/Cas9 delivery approach in plants still faces two obstacles. First, most DNA or RNA positive viruses such as TRV (tobacco rattle virus) have a small cargo capacity (<1 kb) which cannot fit Cas9 (4.1 kb) in the vector; therefore, such viral vectors have to be delivered via a Cas9 overexpression line (OE). Moreover, most of the viral vectors have a low transmitting rate in meristem and germline cells, which requires a somatic cell regeneration step to obtain stable transgenic lines. As a result, two recently developed viral vectors have overcome each of these limitations (Cody and Scholthof, 2019).

The mRNA of the *FT* gene (*Flowering Locus*) can move from the vascular tissue to the apical meristem and can promote cell-cell mobility when fused with other RNA sequences (Mathieu et al., 2007; Ellison et al., 2020). A mobile gRNA targeting phytoene desaturase (*PDS*) fused with the *FT* sequence was cloned into the TRV vector and was injected into Cas9 OE tobacco lines (Ellison et al., 2020). Somatic mutations of *PDS* were successfully transmitted from locally infected leaf tissue to the upper meristem. More importantly, seeds of the T1 mutants were planted and 65% of the T2 generation inherited the mutation. Furthermore, three mobile gRNAs were assembled together to target two tobacco genes (*PDS* and *AGAMOUS*), and ∼30% of the progeny inherited mutations corresponding to the three gRNAs (Ellison et al., 2020).

To generate DNA-free CRISPR/Cas9 edited plants, Ma et al. (2020) recently engineered an RNA negative-strand virus, sonchus yellow net rhabdovirus (SYNV), which was the first reported vector to include the entire CRISPR/Cas9 cassette. This vector was first used to target *GFP* and achieved up to a 90% mutation rate. They went on to target three tobacco genes, *PDS*, *RNA-dependent RNA polymerase 6* (*RDR6*), and *suppressor of gene silencing 3* (*SGS3*) using a tRNA-gRNA multiplexing approach and achieved a 40–90% mutation rate. Although SYNV seems to be an ideal choice to achieve DNA-free gene editing, it can only infect somatic cells and cannot be passed on to the next generation without going through the somatic cell regeneration (Ma et al., 2020).

## Knock-In Genes vs. Knock-Out Genes Using CRISPR/Cas9 in Plants

When CRISPR/Cas9 generates a DSB, it can be repaired by either NHEJ or homologous-directed repair (HDR) with the former being the primary repair pathway in somatic cells and not requiring a donor template (Malzahn et al., 2017). Although rare, HDR can achieve precise gene repair, thus it is often used for precise gene targeting (GT or knock-in genes) and gene replacement (Huang and Puchta, 2019). To perform GT, a gene to be inserted, referred to as the donor template, is flanked by the two homologous arms. Homologous arms contain the same sequences (∼500 bp) adjacent to the GT site (Rozov et al., 2019). The efficiency for knock-out genes by CRISPR/Cas9 can reach 50–100% compared to knock-in genes that is often less than 10%. Moreover, the cloning steps and design of CRISPR/Cas constructs for knock-in genes can be more difficult than for knock-out genes.

It has been reported that HDR favors linearized donor sequences over circular donor sequences, thus target sequences have been inserted before the two homologous arms so that the donor DNA can be released by nucleases (Song and Stieger, 2017; Li et al., 2019). It is believed that the copy number and accessibility of the donor template are rate-limiting factors for HDR (Zhang et al., 2019). Geminivirus, a common plant virus, can generate large numbers of replicons by rolling circle replication. By using a geminivirus-based replicon system to deliver donor templates, the GT rate can be increased 10 to 100 fold in plants (Čermák et al., 2015; Cunningham et al., 2018; Dahan-Meir et al., 2018; Demirer et al., 2019). To make the donor templates more accessible for Cas, donor DNA and Cas have been incorporated into RNP (ribonucleoprotein) complexes and delivered to protoplasts in several plant species including Arabidopsis, tobacco, lettuce, and rice (Ma et al., 2020). The RNP approach has also been used to generate DNA-free knock-out mutants (Woo et al., 2015; Baek et al., 2016; Malnoy et al., 2016). Because biotin and streptavidin (Avidin) form a strong non-covalent linkage, fusing a biotin tag with the donor template and an Avidin tag to Cas helps to better recruit biotinylated donor DNA (Ma et al., 2017). Unlike NHEJ that happens in the cell cycle except for meiosis, HDR happens only during G2/S phase (Orthwein et al., 2015). Therefore, increasing Cas9 activity during meiosis may increase the GT rate through HDR. One study compared the GT rate using several germline cell promoters to drive Cas9 in Arabidopsis and found the egg-cell and the early embryo promoter DD45 (EC1.2) achieved the highest GT (Miki et al., 2018). Interestingly, the investigators were only able to achieve GT by sequentially transforming the gRNA and the donor template into the Cas9 transgenic lines driven by the different promoters and not by transforming the three elements together into non-transgenic lines (Miki et al., 2018).

## Current Efforts Using CRISPR/Cas9 to Study Plant Cell Wall-Related Gene Families

### Utilizing CRISPR/Cas9 to Study Lignin Biosynthesis

Poplar and switchgrass are two important bioenergy crops. Both species are polyploid and outcross with a high frequency of single nucleotide polymorphisms (SNPs) that impede efficient and specific gene editing (Carroll and Somerville, 2009; Okada et al., 2010). However, the multiplexing capacity and specificity of the CRISPR/Cas9 system has been utilized to target genes involved in lignin biosynthesis in these two species.

One study successfully edited two homologous genes (*4CL1* and *4CL2*) in poplar (Zhou et al., 2015). Both *4CL1* and *4CL2* belong to the 4-coumarate: CoA ligase (*4CL*) gene family and are responsible for lignin and flavonoid biosynthesis, respectively (Hu W.J. et al., 1998; Harding et al., 2002). Disruption of *4CL1* lowered the syringyl-to-guaiacyl (S:G) monolignol ratio, resulted in a 23% decrease of the lignin content, and had a slight reduction of condensed tannins (CT), which is a flavonoid derivative, suggesting that some gene redundancy is present between *4CL1* and *4CL2*. Knocking out *4CL2* resulted in a 50–90% reduction of CT only in the roots and 30% less chlorogenic acid in leaves. In the *4cl1* mutants, no off-target editing occurred, including in the *4CL5* gene, which differs by only 1 bp from the gRNA sequence targeting *4CL1* (Zhou et al., 2015; Liu et al., 2016a; Table 1). Further characterization of the *4cl1* mutants found that they contained more caffeic acid, which is a substrate for 4CL5. In addition, the *4cl1* mutants showed reduced expression of ferulate-5-hydroxylase (F5H), a key gene in the S-lignin biosynthesis pathway, and elevated expression of *caffeoyl-CoA O-methyltransferase1* that is involved in G-lignin biosynthesis, suggesting that the reduced S-lignin production in *4cl1* mutants came with a compensatory effect in G-lignin biosynthesis (Tsai et al., 2020). Similar to poplar, switchgrass is a tetraploid species that contains three homologous *4CL* genes, namely *Pv4CL1*, *Pv4CL2*, and *Pv4CL3* (Park et al., 2017). Four out of thirty-nine transgenic lines were edited in one or more of the four alleles of the *Pv4CL1* gene at a single target site. Suppression of *Pv4CL1* showed reduced cell wall thickness, up to 30% less lignin, 7–11% more glucose and 23–32% more xylose (Park et al., 2017; Table 1).

**TABLE 1:** Examples of plant cell wall related genes edited by CRISPR/Cas9.

**Gene(s) edited**	**Mutation type**	**Number of gRNA and number of genes targeted**	**Species**	**Mutant phenotype**
*4CL1* (*4-coumarate: CoA ligase 1*)	Biallelic mutation	One gRNA targeting one gene	Poplar	Lower syringyl-to-guaiacyl (S: G), 23% reduction of lignin, more extractable polysaccharide in the chlorite fraction of the cell wall; an increase of caffeic acid; upregulation of *caffeoyl-CoA O-methyltransferase1* and downregulation of *5-hydroxylases* (*F5Hs*) (Zhou et al., 2015; Tsai et al., 2020).
*4CL2* (*4-coumarate: CoA ligase 2*)	Biallelic mutation	One gRNA targeting one gene	Poplar	50–90% reduction of condensed tannins (CT) in roots; 30% reduction of chlorogenic acid (Zhou et al., 2015).
*OsCALd5H* (*coniferaldehyde 5-hydroxylase*)	Monoallelic and biallelic mutations	Multiple gRNAs targeting one gene	Rice	More lignin in leaf sheath and more arabinoxylan in culm cell walls (Takeda et al., 2019).
*BcFLA1* (fasciclin-like AGPs 1)	Biallelic mutation	Two gRNAs targeting one gene	*Brassica carinata*	Reduction in root hair length under Pi deficient conditions (Kirchner et al., 2017, 2018).
*GLCAT14A, GLCAT14B*, and *GLCAT14C* (*glucuronic acid transferases*)	Biallelic mutations	Three to four gRNAs targeting three genes	Arabidopsis	All *glcat* mutants showed less calcium binding on their AGPs. The *glcat14a glcat14b* and *glcat14a glcat14b glcat14c* showed a delay in seed germination, impaired trichome and root hair growth, and less adherent seed mucilage (Zhang et al., 2020).
*OsXYN1* (*endo-1,4-*β*-xylanase*)	Biallelic mutation	One gRNA targeting one gene	Rice	Dwarf, thinner stems, leaf tip necrosis; less lignin content; less water intake; downregulation of genes in xylan and lignin biosynthesis pathways; upregulation of genes in the aquaporin water channel pathway (Tu et al., 2020).
*PL* (*pectate lyase*)	Biallelic mutation	One gRNA targeting one gene	Tomato	Firmer inner and outer pericarp; higher juice and paste viscosity (Wang D. et al., 2019).
*PG2a* (*polygalacturonase 2a*)	Biallelic mutation	One gRNA targeting one gene	Tomato	Higher juice and paste viscosity; Fruit color change delay (Wang C. et al., 2019).
*TBG4* (*beta-galactanase*)	Biallelic mutation	One gRNA targeting one gene	Tomato	More separation of the intracellular spaces and larger fruit size; fruit color change delay (Wang D. et al., 2019).
*Bra003491, Bra007665, Bra014410*(pectin methylesterase)	Biallelic mutation	One gRNA targeting one to three genes	*Brassica campestris*	Not reported (Xiong et al., 2019).
*CESA3* (*cellulose synthase gene 3*)	C to T conversion	One gRNA targeting one gene	Rice	Conferring C17 and isoxaben double herbicide resistance (Hu Z. et al., 2019).
*PtoMYB156*	Partial deletion	Three gRNAs targeting one gene	Poplar	Thicker SCW and elevated expression of genes involved in SCW biosynthesis (Yang L. et al., 2017).
*EVE* (*enlarged vessel element*)	Biallelic mutation	One gRNA targeting one gene	Poplar	Less vessel elements and reduction in vessel area (Ribeiro et al., 2020).
*OsSND2 (secondary NAC domain 2)*	Biallelic mutation	Two gRNAs targeting one gene	Rice	Less cellulose content, down-regulation of CESAs, thinner cell wall (Ye et al., 2018).
*OsIDD2 (interminate domain 2)*	Biallelic mutation	One gRNA targeting one gene	Rice	Slight increase in lignin content and more phloroglucinol staining in leaf vascular tissue (Huang et al., 2018).

Rice is another important biomass crop species for biofuel production. One key enzyme in the lignin biosynthesis pathway is coniferaldehyde 5-hydroxylase (CALd5H), which influences the S:G ratio (Boerjan et al., 2003; Vanholme et al., 2008; Takeda et al., 2017). In addition, grass species also contain γ-*p*-coumaroylated G/S monolignols. Three sgRNAs (a, b, and c) were selected by the CRISPR-P program and assembled into a single construct to target different loci of the *OsCALd5H* gene in rice (Takeda et al., 2019; Table 1). Both sgRNA-a and sgRNA-c achieved 83–100% targeting rates, whereas no editing was detected for sgRNA-b in the T0 generation. Homozygous Cas9-free T1 plants were also generated from *OsCALd5H*-KO-a and *OsCALd5H*-KO-c lines. Both mutant lines contained more lignin in leaf sheaths and more arabinoxylan in culm cell walls compared to WT rice. Moreover, 2D NMR analysis showed a substantial increase in G lignin and a reduction of S lignin, but the γ-p-coumaroylated G/S ratio was not affected, suggesting a dominant role of OsCALd5H in modulating non-γ-*p*-coumaroylated sinapyl alcohol (Takeda et al., 2019).

### Using CRISPR/Cas9 to Study Plant Cell Wall Protein Function

The AGPs are a family of heavily glycosylated cell wall HRGPs found throughout the plant kingdom (Schultz et al., 2002; Showalter et al., 2010; Nguema-Ona et al., 2014). Although 85 AGPs were identified in Arabidopsis, only a few AGP mutants have been characterized due in part to gene redundancy within the family (Showalter et al., 2010). RNA interference (RNAi) and CRISPR/Cas9 are two key molecular techniques that would eliminate or suppress the expression of multiple AGP genes (Levitin et al., 2008; Li et al., 2010; Hou et al., 2016; Pereira et al., 2016; Moreira et al., 2020; Zhang et al., 2020). FLAs (fasciclin-like AGPs) are a distinct subfamily of AGPs that contain AGP domains as well as fasciclin protein domains that are believed to function in cell adhesion (Johnson et al., 2003). One study performed in *B. carinata* discovered that *BcFLA* was specifically downregulated in response to inorganic phosphate (Pi) deficient conditions (Kirchner et al., 2018). To study the role of *BcFLA*, two gRNAs were designed to target *BcFLA1*. As there are two alleles (*BcFLA1a* and *BcFLA1b*) that are similar in their sequences, both gRNAs were designed to match the sequence of the *BcFLAa* allele. While the 1st gRNA sequence contained two mismatches to the sequence of *BcFLA1b*, the 2nd gRNA contained four mismatches to *BcFLA1b* including one mismatch in the PAM sequence. A number of gene-editing events were detected and mostly occurred in the 1st gRNA targeting region ranging from 5 to 154 bp deletions in both alleles. Phenotypic analysis of the CRISPR induced *fla1* mutant found its root hairs were ∼50% shorter in response to Pi starvation (Kirchner et al., 2018; Table 1). As the genome of *B. carinata* is not fully sequenced, it was not possible to test for possible off-target events. To confirm that the reduced root hair length was caused by the disruption of *BcFLA1*, a gene complementation analysis was performed that expressed a mutated version of *BcFLA1a*_*m*_ under the control of a ubiquitin promoter; this mutant allele encodes the same amino acid but was resistant to CRISPR/Cas9 induced mutation (Kirchner et al., 2017). The *BcFLA1a*_*m*_ transgenic line showed an increase in root hair length compared to the *fla1* mutant, confirming the functional importance of BcFLA1 in root hair elongation (Kirchner et al., 2018).

### Using CRISPR/Cas9 to Study Plant Cell-Wall Associated Enzymes

AGPs are modified by the addition of type II arabinogalactan (AG) polysaccharides, which includes a β-(1,3)-linked galactose backbone which is modified with the addition of multiple β-(1,6)-galactan side chains which include galactose (Gal), arabinose, fucose, glucuronic acid (GlcA), rhamnose, and xylose residues (Showalter, 2001; Showalter and Basu, 2016a). The backbone and sidechains are synthesized by the step-wise action of a set of specific glycosyltransferases, which act mainly in the Golgi to specifically add each of these sugars to particular locations in the AG polysaccharide. Thus, one approach to reveal functional roles of the sugar decorations on AGPs is to knock out these glycosyltransferase (GT) genes and examine phenotypic changes in the resulting mutants (Basu et al., 2013, 2015a,b; Liang et al., 2013). However, gene redundancy present in most GT families often results in single mutants with no observable phenotypic differences from WT. Moreover, it is difficult and time consuming to create higher order mutants and disrupt multiple genes simultaneously by genetically crossing only T-DNA mutants (Ogawa-Ohnishi and Matsubayashi, 2015; Showalter and Basu, 2016b). Most recently, we have applied a CRISPR/Cas9 approach to edit three glucuronic acid transferases (GLCATs) simultaneously in Arabidopsis (Zhang et al., 2020; Table 1). These GLCATs function in adding GlcA onto AGPs; GlcA is the only negatively charged sugar molecule on AGPs and is proposed to be the key sugar molecule enabling AGPs to bind extracellular calcium (Lamport and Várnai, 2012, 2013; Knoch et al., 2013; Dilokpimol and Geshi, 2014; Lamport et al., 2014). In our work, we found a substantial reduction in AGP calcium binding in all the CRISPR mutant lines compared to WT. Furthermore, this CRISPR/Cas9 multiplexing approach was essential in identifying the redundant roles of two of these physically linked genes, namely *GLCAT14A* and *GLCAT14B*, in regulating seed germination, root hair growth, trichome development, and plant reproduction (Zhang et al., 2020).

Xylan is a main component in the secondary cell wall that contributes to mechanical strength and cell wall recalcitrance. The structure of xylan consists of a β-1,4-linked xylopyranosyl (Xyl) backbone and often decorated by α-L-arabinopyranose (Ara*f*) as a single unit and sometimes substituted with 4-*O*-methyl-α-D glucuronic acid (GlcA) (Darvill et al., 1980). Previous studies identified two rice mutants, *ss1* and *ss2*, that exhibited dwarf, thinner stems, and leaf tip necrosis phenotypes (Tu et al., 2015). A follow-up study found that these two mutants contain point mutations in the gene named *OsXYN1*, which encodes an endo-1,4-β-xylanase (Tu et al., 2020). To confirm the role of OsXYN1, two *OsXYN1* CRISPR mutant lines were produced that contained 1 and 2 bp deletions, respectively (Tu et al., 2020; Table 1). As expected, both *OsXYN1* mutants demonstrated similar phenotypes to *ss1* and *ss2*. Furthermore, the *ss* mutants contained less lignin and downregulated genes related to xylan and lignin biosynthesis (Tu et al., 2020). Moreover, the *ss* mutants were likely to wilt under sunlight and demonstrated inefficient water uptake, which was caused by having a thinner middle lamella compared to WT. As a trade-off, genes in the aquaporin water channel pathway were found to be upregulated in the *ss* mutants (Tu et al., 2020).

Manipulating enzymes in the pectin degradation pathway can potentially enhance the post-harvest life of fruits such as tomatoes (Kitagawa et al., 2005). A recent study successfully edited *pectate lyase (PL)*, *polygalacturonase 2a (PG2a*), and β*-galactanase (TBG4)* to reveal their functions in pectin degradation and fruit ripening in tomato (Wang D. et al., 2019; Table 1). The PL CRISPR line showed a firmer inner and outer pericarp. Both the *PL* and *PG2a* CRISPR lines showed higher juice and paste viscosity. The *TBG4* CRISPR lines exhibited more separation of the intracellular spaces and larger fruit size, whereas *TBG4* and *PG2a* CRISPR lines also showed a delay in fruit color changes during ripening (Wang D. et al., 2019). Another recent study also highlighted multi-gene targeting of three pectin-methylesterase genes (*Bra003491*, *Bra007665*, *Bra014410*) using a single gRNA and achieved a 20–56% mutation rate in *Brassica campestris* (Xiong et al., 2019; Table 1).

The CRISPR/Cas9 mediated genome editing approach has emerged as a powerful tool to modify cell wall biosynthesis pathways in several plant species. Recently, the base-editing property of CRISPR/Cas9 was exploited to generate double herbicide resistant rice plants (Hu Z. et al., 2019). C17 is a newly identified growth inhibitor that can disrupt cellulose biosynthesis by directly acting on CESA1 or CESA3. However, a C17 resistant mutant line has been identified in Arabidopsis that contained a C to T point mutation in *CESA3*. By transforming a BE3-CESA3^*S*983*F*^ CRISPR/Cas9 base editor system with cytidine deaminase into another isoxaben resistant mutant (*irx2-1*) background, 9 out of 2,000 transgenic lines conferred C17 and isoxaben resistance, including seven chimeric mutants and two homozygous and Cas9-free mutant lines (Hu Z. et al., 2019; Table 1).

### Using CRISPR/Cas9 to Study Transcription Factors in Secondary Cell Wall Biosynthesis

Secondary cell walls (SCWs) are found in vessel and fiber cells. The SCW biosynthesis pathway is mainly regulated by two kinds of transcription factors (TFs), namely NAC and MYB, with the former serving as the master switch for the latter. A 19 bp secondary cell wall NAC-binding element (SNBE) and a 7 bp SCW MYB-responsive element (SMRE) are the binding sequences for NAC and MYB for downstream gene regulation, respectively (Zhong et al., 2010; McCarthy et al., 2011; Zhong and Ye, 2015). To manipulate SCW biosynthesis, a CRISPR/Cas9 genome editing approach was utilized to target these two TFs as well as their target genes.

In poplar, there are at least 192 putative R2R3 MYB TFs (Wilkins et al., 2009). CRISPR/Cas9 was used to reveal the function of a R2R3 MYB repressor named *PtoMYB156* (Yang L. et al., 2017; Table 1). An overexpression line of *PtoMYB156* showed thinner SCWs in xylem fibers, a reduction cellulose, lignin, and xylose content. Moreover, three gRNAs corresponding to sequences in the first exon were used to target *PtoMYB156* (Yang L. et al., 2017). PCR-based analysis found three CRISPR lines (Line 5, Line 7, and Line 12) contained partial deletions between gRNA2 and gRNA3. Moreover, sequencing results showed 12 out of 25 clones (48%) of Line 5 contained a deletion between gRNA2 and gRNA3, indicating this line is likely to be a heterozygous mutant. Gene-editing of *PtoMYB156* resulted in more lignin in the phloem fibers coupled with increased expression of the genes involved in SCW biosynthesis (Yang L. et al., 2017).

CRISPR/Cas9 gene editing was also recently used to identify the roles of a previously uncharacterized gene named *ENLARGED VESSEL ELEMENT* (*EVE*), which was initially identified using QTL analysis in poplar (Ribeiro et al., 2020). A CRISPR/Cas9 *eve* mutant was created and exhibited fewer vessel elements and a reduction in vessel area, whereas an EVE-overexpression (OE) line exhibited 129% larger vessels compared to WT. The *eve* and EVE-OE lines also showed a 23% decrease and a 39% increase of root vessel diameter, respectively. Moreover, the larger vessel elements in the EVE-OE line also demonstrated increased K^+^ uptake compared to that of *eve* mutant and WT. The authors argued that the absence of changes in K^+^ uptake in the *eve* mutant was due to compensation effects by other potassium transporters (Ribeiro et al., 2020; Table 1). By fusing the promoter region of EVE with the LUCIFERASE enzyme and in the presence of several TFs, the transcription of *LUCIFERASE* a significant increase in the presence of the secondary cell wall-associated NAC domain protein, SND1. Consistently, a 19-bp SNBE was also found in the promoter region of EVE that can bind to SND1 (Ohtani et al., 2017).

OsSND2, which is a NAC, was identified as the activator for *OsMYB61* by yeast one-hybrid screening and transactivation analysis (Ye et al., 2018). Two transgene-free, homozygous rice mutants named *snd2-c1* and *snd2-c2* were generated by cloning two gRNAs corresponding to the first exon of *OsSND2* in one gene construct (Ye et al., 2018; Table 1). The *snd2* mutants phenocopied the WT rice but contained significantly less cellulose and had thinner cell walls in sclerenchyma cells. In contrast, *OsSND2*-OE plants were semi-dwarf, displayed significant leaf rolling phenotypes, had greater cellulose content. As expected, expression levels of several *CESAs* and R2R3-type *MYBs* were down-regulated in the *snd* mutants and up-regulated in *OsSND2*-OE lines (Ye et al., 2018).

In rice, 123 TFs were identified to be involved in SCW biosynthesis based on co-expression network analysis (Hirano et al., 2013). Among them, one is a zinc-finger TF named INTERMINATE DOMAIN 2 (OsIDD2), which can negatively regulate SCW formation (Huang et al., 2018). The OsIDD2-OE lines exhibited a clear dwarf phenotype as well as brittle leaves. This line also contains 50% less lignin compared to WT rice. However, *osidd2* CRISPR mutants showed no clear phenotypes and had only a slight increase in lignin content (Table 1). Phloroglucinol staining of the *osidd2* mutant showed darker staining of leaf vascular bundles compared to WT, whereas little staining was observed in the OsIDD2-OE lines. Transient expression analysis using a firefly luciferase (fLUC) reporter found that OsIDD2 negatively regulates expression of *cinnamyl alcohol dehydrogenase 2 and 3* (*CAD2* and *CAD3*) and *sucrose synthase 5* (*SUS5*) (Huang et al., 2018).

## Conclusions

Efforts have been made over the past decades to identify enzymes involved in cell wall biosynthesis. For cell wall researchers, understanding the biochemical and physiological properties of different cell wall components is crucial for generating genetically engineered plants with desired cell wall traits for plant growth and commercial applications. Given the large variation in carbohydrate and linkage types, we have yet to fully understand the complexity and interactions associated with cell wall structure. Moreover, gene redundancy for many genes encoding cell wall biosynthesis enzymes has made it challenging to elucidate the biological function of specific cell wall components by conventional methods. In this review, we have shown that the specificity and the multiplexing features of CRISPR/Cas9 makes it an ideal tool to uncover biological functions of genes, and particularly gene families demonstrating functional gene redundancy, associated with the biosynthesis of plant cell wall components. Moreover, the shorter timeframe from genotype to phenotype makes CRISPR/Cas9 mutagenesis particularly valuable and appealing to generate higher-order mutants to discover gene functions for many cell wall-related gene families. Finally, while we have largely focused on cell wall biosynthesis here, this CRISPR/Cas9 approach is equally applicable to other genes whose products function in modifying and/or degrading the plant cell wall.

## Author Contributions

YZ wrote the manuscript. AMS proposed and assisted in writing the manuscript. Both authors contributed to the article and approved the submitted version.

## Conflict of Interest

The authors declare that the research was conducted in the absence of any commercial or financial relationships that could be construed as a potential conflict of interest.

## Footnotes

1 https://www.genome.arizona.edu/crispr/

2 http://www.e-crisp.org/E-CRISP/

3 https://chopchop.cbu.uib.no/

4 http://crispr.tefor.net/

5 http://crispr.hzau.edu.cn/CRISPR2/
